# Application Value of Enhanced CT Imaging Features in Predicting Vessels Encapsulating Tumor Clusters (VETC) Positivity in Hepatocellular Carcinoma

**DOI:** 10.2174/0115734056361565250530050624

**Published:** 2025-06-10

**Authors:** Qianjiang Ding, Xi Deng, Jingfeng Huang, Ruixue Zhang, Ting Liu, Jianhua Wang, Yutao Wang

**Affiliations:** 1 Department of Radiology, The First Affiliated Hospital of Ningbo University, Ningbo, Zhejiang 315010, China; 2 Department of Pathology, Ningbo Clinical Pathology Diagnosis Center, Ningbo, Zhejiang 315021, China; 3 Department of Ultrasound, The First Affiliated Hospital of Ningbo University, Ningbo, Zhejiang 315010, China; 4 Department of Radiology, The First Affiliated Hospital of Xiamen University,Xiamen, Fujian 361000,China; 5 Department of Radiology, Ningbo Ninth Hospital, Ningbo, Zhejiang 315020, China

**Keywords:** Hepar, Hepatocellular carcinoma, Vessels Encapsulating Tumor Clusters (VETC), VETC-positive, Computed Tomography(CT), Enhanced CT

## Abstract

**Background:**

VETC-positive has emerged as a novel predictor of HCC for poor prognosis. Enhanced CT is one of the most common diagnostic methods, which can indicate VETC positivity, providing important evidence for the diagnosis and treatment of VETC-positive HCC.

**Objective:**

The objective of this study is to investigate the clinical and preoperative enhanced CT imaging characteristics and diagnostic value of VETC-positive hepatocellular carcinoma (HCC) patients.

**Methods:**

A retrospective analysis was conducted on the clinical, pathological, and imaging data of 53 HCC patients from the First Affiliated Hospital of Ningbo University between June 2019 and September 2022. According to pathological results, patients were categorized into 11 VETC-positive and 42 VETC-negative cases. Observational parameters included: (1) Clinical indicators: gender, age, history of hepatitis B virus infection, preoperative AFP, TNM staging, and preoperative biochemical and coagulation laboratory tests, including Alb, AST, ALT, TBil, DB, PT, TT, and INR. Additionally, pathological results such as histological grading, microvascular invasion (MVI), satellite nodules, neural invasion, and postoperative recurrence were analyzed. (2) Preoperative enhanced CT observational indicators: maximum tumor diameter, intrahepatic growth, irregular tumor margins, peritumoral hepatic parenchymal enhancement, mosaic structure, non-ring-like arterial phase hyperenhancement, marked heterogeneous enhancement, non-peripheral washout, absence of enhancing capsule, enhancing/clear capsule, intratumoral arteries, intratumoral necrosis, along with measurement of unenhanced CT values and enhanced CT values at various phases, calculating enhancement ratios (enhancement ratio = enhanced CT value - unenhanced CT value / unenhanced CT value).

Quantitative data were expressed as mean ± standard deviation (x̅±s), with intergroup comparisons conducted using the *t-*test; categorical variables were compared using the *χ^2^* test or *Fisher's* exact test. Multivariate analysis employed stepwise regression for logistic regression, incorporating clinical and imaging characteristics into the logistic regression equation. Based on logistic regression results, receiver operating characteristic (ROC) curves were plotted, calculating the area under the curve (AUC), sensitivity, specificity, and their 95% confidence intervals (CI). Analysis on survival was performed using Kaplan-Meier methods and log-rank tests, aiming survival curves.

**Results:**

(1) Clinical characteristics of VETC-positive versus VETC-negative patients: Preoperative AFP levels showed statistical significance (P=0.037), while no significant differences were observed in gender, age, Alb, TB, DB, AST, ALT, PT, TT, and INR between VETC-positive and VETC-negative patients (P>0.05). (2) Enhanced CT imaging features of VETC-positive versus VETC-negative patients: Intratumoral necrosis showed statistical significance (P<0.05), with intratumoral arteries being 63.6% (7/11) in the positive group compared to 42.9% (18/42) in the negative group. No significant differences were found in maximum tumor diameter, irregular tumor margins, peritumoral hepatic parenchymal enhancement, mosaic structure, non-ring-like arterial phase hyperenhancement, marked heterogeneous enhancement, non-peripheral washout, absence of enhancing capsule, enhancing capsule, intratumoral arteries, as well as unenhanced CT values and enhanced CT values at various phases, arterial phase enhancement ratio, portal phase enhancement ratio, and delayed phase enhancement ratio (P>0.05). (3) Multivariate analysis influencing VETC positivity: Arterial phase CT values (HU) (OR=0.937, P=0.029), intratumoral arteries (OR=9.452, P=0.021), and intratumoral necrosis (OR=0.013, P=0.003) were identified as independent risk factors for VETC positivity (Odds Ratio=0.937, 9.452, 0.013, 95% CI=0.883-0.993, 1.4-63.823, 0.001-0.223, P<0.05). The AUC of VETC was 0.863 (95% CI: 0.728-0.997), with a sensitivity of 81.8% and specificity of 88.1%. (4) Postoperative early tumor recurrence in VETC-positive and VETC-negative patients: All 53 patients were followed up, with an average tumor recurrence time of 11 (4-20) months, showing significant differences (P<0.05).

**Conclusion:**

As one of the routine and preferred methods for HCC examination, enhanced CT plays a pivotal role in diagnosis, staging, and post-treatment evaluation. Combining preoperative enhanced arterial phase CT values, intratumoral arteries, and intratumoral necrosis can highly indicate VETC positivity.

## 1 INTRODUCTION

The latest report from the National Cancer Center indicates that hepatocellular carcinoma (HCC) remains the fourth most prevalent malignancy and the second leading cause of cancer mortality in China [1, 2]. Postoperative 5-year recurrence and metastasis rates of HCC remain as high as 40% to 70%, possibly related to pre-existing early micro-metastases or micrometastatic foci [1, 3]. Vessels Encapsulating Tumor Clusters (VETC) has emerged as a novel predictor of tumor aggressiveness and poor prognosis, encapsulating tumor nests with endothelial cells to form independent tumor cell clusters. These clusters disseminate through the bloodstream, establishing metastases in target organs more effectively than traditional epithelial-mesenchymal transition pathways, thereby increasing tumor recurrence and metastasis rates [3]. Studies by Renne SL [4] have also demonstrated that VETC-positive HCC exhibits earlier postoperative recurrence and shorter overall and disease-free survival compared to VETC-negative HCC. Therefore, preoperative assessment of VETC status in HCC is of significant clinical importance.

Dynamic enhanced CT and multi-parameter MRI are the primary imaging modalities for definitive HCC diagnosis [1], playing an essential role in predicting VETC positivity. Existing studies have predominantly focused on gadoxetate disodium (Gd-EOB-DTPA) enhanced MRI imaging characteristics and radiomics for predicting VETC [5-7], with limited reports on enhanced CT features [8]. Hence, this study aims to enrich the conventional imaging characteristics used for preoperative assessment of VETC-positive HCC based on preoperative clinical and enhanced CT data, offering valuable screening strategies for stratifying HCC risk populations and selecting appropriate treatment plans [9].

## 2 MATERIALS AND METHODS

### 2.1 General Data

This is a case-control study. A retrospective analysis was conducted on the clinical, pathological, and imaging data of 53 HCC patients treated at the First Affiliated Hospital of Ningbo University between June 2019 and September 2022. The cohort consisted of 46 males and 7 females, with an average age of 60±9 years. Postoperative histopathological examination confirmed 11 VETC-positive and 42 VETC-negative cases. This study was approved by the Ethics Committee of the First Affiliated Hospital of Ningbo University, approval number 2024-188RS, with patient consent waived.

### 2.2 Inclusion and Exclusion Criteria

#### 2.2.1 Inclusion Criteria

(1) Complete preoperative enhanced CT data, with less than 2 weeks between imaging and surgery. (2) Histopathological confirmation of HCC post-surgery. (3) Complete clinical and pathological data.

#### 2.2.2 Exclusion Criteria

(1) Preoperative antitumor treatment. (2) Unclear enhanced CT images and/or missing clinical pathological data.

### 2.3 Enhanced CT Examination Method

Enhanced scans were performed using a Toshiba Aquilion 64-slice CT or Aquilion One 320-slice volume CT. Scans were conducted with the patient in a supine position, feet first, from the diaphragm to the iliac crest level. A dual-channel high-pressure injector was used to administer the contrast agent *via* the median cubital vein at a rate of 4.5-5 ml/s. First, approximately 70 ml of contrast agent was injected, followed by 20 ml of saline at the same rate. The contrast agent used was iohexol (GE Healthcare iohexol injection, 350 mg I/ml). Volumetric scans were performed in the arterial phase (35-40s post-injection), portal phase (60-70s post-injection), and delayed phase (180s post-injection). Reconstructed images had a slice thickness of 2 mm and an interval of 0 mm.

### 2.4 Image Evaluation Method

Enhanced CT images were reviewed independently by two radiologists with over seven years of experience in abdominal imaging. In cases of disagreement, a third senior radiologist with over ten years of experience reviewed the images and reached a consensus through discussion. Pathological results were reviewed by two pathologists with over ten years of experience in histopathological diagnosis, who independently evaluated CD34 immunohistochemical staining of postoperative tumor tissues for VETC, reaching a consistent diagnosis.

### 2.5 Observational Parameters and Evaluation Standards

#### 2.5.1 Observational Indicators

(1) Clinical characteristics of VETC-positive versus VETC-negative patientsincluded gender, age, history of hepatitis B virus infection, preoperative AFP, TNM staging, preoperative biochemical and coagulation laboratory tests (Alb, AST, ALT, TBil, DB, PT, TT, and INR), and pathological results (histological grading, microvascular invasion, satellite nodules, neural invasion).

(2) Enhanced CT imaging features of VETC-positive versus VETC-negative patients were based on literature [8] and the Liver Imaging Reporting and Data System (LI-RADS) for diagnosing HCC. The enhanced CT imaging observational indicators for VETC-positive and VETC-negative patients included maximum tumor diameter, intrahepatic growth, irregular tumor margins, peritumoral hepatic parenchymal enhancement, mosaic structure, non-ring-like arterial phase hyperenhancement, marked heterogeneous enhancement, non-peripheral washout, absence of enhancing capsule, enhancing/clear capsule, intratumoral arteries, intratumoral necrosis, as well as unenhanced CT values and enhanced CT values at various phases, and the calculation of enhancement ratios (enhancement ratio = enhanced CT value - unenhanced CT value / unenhanced CT value).

(3) Multifactorial Analysis Influencing VETC Positivity in HCC Patients: Clinical and imaging characteristic variables were included in the multivariate analysis.

(4) Postoperative Tumor Recurrence in VETC-Positive and VETC-Negative Patients: This included the number of patients followed up, follow-up duration, and tumor recurrence time.

#### 2.5.2 Evaluation Criteria

(1) Enhanced CT imaging features include: Enhanced CT characteristics encompass: (1) Maximum tumor diameter; (2) Unenhanced CT values and enhanced CT values at various phases, as well as enhancement ratios; (3) Non-ring-like arterial phase hyperenhancement, non-peripheral washout, and enhancing capsule are primary indicators for diagnosing HCC in the Liver Imaging Reporting and Data System (LI-RADS); (4) Non-enhancing capsule, mosaic structure, and rim-like enhancement are auxiliary indicators for diagnosing malignant lesions in LI-RADS; (5) Irregular tumor margins are defined as nodular elevation, multinodular confluence, or infiltration; (6) Peritumoral enhancement in the arterial phase is defined as wedge-shaped or irregular enhancement in the hepatic parenchyma around the tumor in the arterial phase; (7) Intratumoral arteries are defined as discontinuous or tortuous linear enhancement within the tumor in the arterial or portal phase; (8) Capsule: a complete capsule is defined as a uniform border around most or all of the tumor, appearing as an enhancing margin in the portal or delayed phase; a non-enhancing capsule refers to a low-density ring around the lesion in the arterial or portal phase; (9) Tumor necrosis or ischemia is defined as non-enhancing or low-enhancing areas accounting for ≥20% in the largest cross-sectional area of the tumor on enhanced scans at various phases.

(2) Postoperative histopathological examination evaluation: According to the literature [3, 10],VETC positivity is defined by the presence of VETC structures in tumor tissue sections stained with CD34 immunohistochemistry. VETC structures are defined as sinusoidal tumor blood vessels encapsulating tumor cells in a cluster-like formation on CD34 immunohistochemically stained tumor tissue sections.

(3) Postoperative recurrence is defined by evidence of AFP and imaging examinations (ultrasound, CT, or MRI) or histopathological examination confirming tumor recurrence and metastasis. Recurrence time is defined as the interval from surgery to the first recurrence or the end of follow-up.

### 2.6 Follow-up

Follow-up was conducted through shared access to health records, using the “cloud archive” to gather information on tumor recurrence. The follow-up period concluded in January 2024.

### 2.7 Statistical Analysis

Data were analyzed using SPSS 22.0 statistical software. Quantitative data were expressed as mean ± standard deviation (x̅±s), with intergroup comparisons conducted using the *t*-test. Categorical variables were compared using the *χ^2^* test or *Fisher's* exact test. Multivariate analysis was performed using stepwise regression for logistic regression, incorporating clinical and imaging characteristics into the logistic regression. Based on logistic regression results, ROC, were plotted to calculate the AUC, sensitivity, specificity, and their 95% CI. Survival analysis was conducted using the Kaplan-Meier method and log-rank tests, with survival curves plotted. A P-value of <0.05 was considered statistically significant.

## 3 RESULTS

### 3.1 Clinical Characteristics of VETC-Positive and VETC-Negative Patients

53 patients were included in the study. Preoperative AFP levels exhibited statistically significant differences between VETC-positive and VETC-negative patients (P<0.05). However, no statistically significant differences were observed in gender, age, Alb, TB, DB, AST, ALT, PT, TT, INR, and pathological results between the two groups (P>0.05) (Tables **1** and **2**).

### 3.2 Imaging Characteristics of VETC-Positive and VETC-Negative Patients

**Fig. (1):**
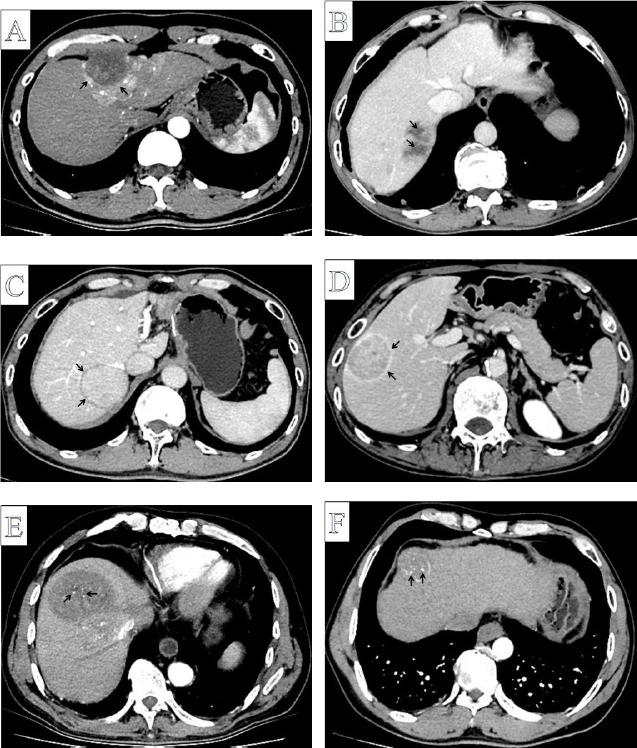
Enhanced CT imaging features. (**A**) Peritumoral hepatic parenchymal enhancement (arrow). (**B**) Intratumoral necrosis (arrow). (**C**) Absence of enhancing capsule (arrow). (**D**) Enhancing/well-defined capsule (arrow). (**E, F**) Intratumoral arteries (arrow).

Comparative analysis of enhanced CT imaging features between VETC-positive and VETC-negative patients revealed statistically significant differences in intratumoral necrosis (P<0.05). Nevertheless, no significant disparities were observed in maximum tumor diameter, irregular tumor margins, peritumoral hepatic parenchymal enhancement, mosaic structure, non-ring-like arterial phase hyperenhancement, marked heterogeneous enhancement, non-peripheral washout, absence of enhancing capsule, enhancing capsule, intratumoral arteries, unenhanced CT values, and enhanced CT values at various phases, arterial phase enhancement ratio, portal phase enhancement ratio, and delayed phase enhancement ratio (P>0.05) (Table **3** and Fig. **1**).

### 3.3 Multivariate Analysis Influencing VETC Positivity

**Fig. (2):**
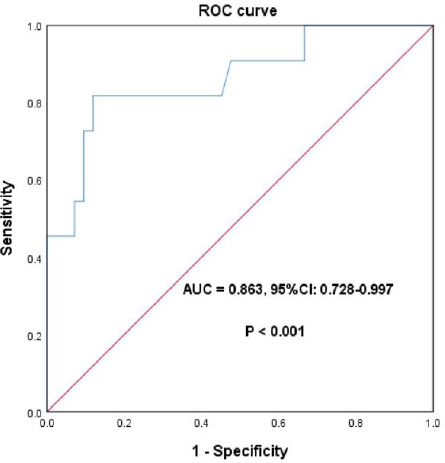
Independent risk factors and roc curve for VETC-positive HCC.

Stepwise logistic regression incorporating clinical characteristics and imaging features demonstrated that arterial phase CT values, intratumoral arteries, and intratumoral necrosis were independent risk factors influencing VETC positivity (P<0.05). The logistic regression, comprising arterial phase CT values, intratumoral arteries, and intratumoral necrosis, predicted VETC with an AUC of 0.863 (95% CI: 0.728-0.997), a sensitivity of 81.8%, and a specificity of 88.1%. Refer to (Table **4** and Fig. **2**).

### 3.4 Recurrence

**Fig. (3):**
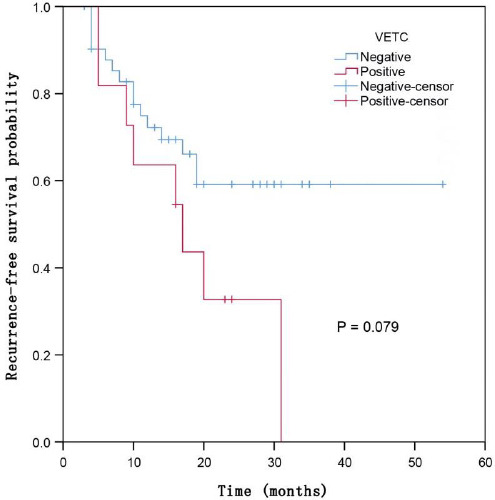
Survival curves for VETC-positive and VETC-negative HCC patients.

By January 2024, all 53 patients were followed up, with an average tumor recurrence time of 11 (4-20) months. The 1-year, 2-year, and 3-year recurrence-free survival rates for the VETC-positive group were 63.6%, 32.7%, and 0%, respectively, while for the VETC-negative group, these rates were 72.2%, 59.2%, and 59.2%, respectively. Chi-square analysis indicated statistically significant differences between the two groups (P<0.05), with survival curves also plotted (P=0.079). Refer to (Fig. **3**).

## 4 DISCUSSION

Since its initial designation by Fang *et al*. in 2015 [3], Vessels Encapsulating Tumor Clusters (VETC) has garnered substantial academic attention as a novel microvascular pattern. VETC-positive HCC is associated with a 1.52-fold increased risk of early recurrence compared to VETC-negative HCC, with earlier recurrence times and shorter overall and disease-free survival [11]. In this study, the postoperative recurrence rate in the VETC-positive group was approximately 72.7% (8/11), significantly higher than the 35.7% (15/42) observed in the VETC-negative group. The survival curves also visually depict shorter recurrence times for VETC-positive patients compared to the VETC-negative cohort. Thus, preoperative evaluation of VETC status in HCC is crucial for prognostication and therapeutic planning.

Research by Yamashita *et al*. [12] demonstrated that VETC-positive HCC patients exhibit aggressive phenotypes, often characterized by higher rates of hepatitis B virus infection and elevated serum alpha-fetoprotein (AFP) levels. In this cohort, 54.5% (6/11) of VETC-positive cases had elevated AFP levels, compared to 28.6% (12/42) in the VETC-negative group, indicating a statistically significant difference. Additionally, VETC-associated studies have shown significant differences in preoperative Alb [13], AST [14], and HBV positivity [15] among VETC-positive patients. However, no definitive clinical indicators have been established for preoperative VETC assessment in HCC patients.

Dynamic enhanced CT and multi-parameter MRI are the preferred imaging modalities for HCC diagnosis, staging, and therapeutic evaluation [1]. Enhanced CT imaging can reflect tumor margins, morphology, capsule size, enhancement patterns, halo sign, invasion of major blood vessels, and presence of distant metastasis. Enhanced CT also allows direct measurement of CT values and other quantitative indicators. Enhanced CT imaging is less affected by respiratory motion and patient cooperation, providing high-quality images that facilitate case selection and exclusion. Despite its advantages, there are limited reports on using enhanced CT features for VETC assessment. Feng *et al*. [8] reported that VETC-positive HCC patients exhibit larger tumor diameters (>5 cm), intratumoral necrosis, intratumoral arteries, and tumor capsules on CT imaging, with tumor size and intratumoral necrosis identified as independent predictors of VETC positivity. Meng *et al*. [8] constructed a model using CT indicators to predict the prognosis of VETC-positive HCC with diameters ≥3 cm, finding that heterogeneous enhancement in the arterial phase (including septal and irregular ring-like enhancement), intratumoral necrosis, and ≥20% hypo-enhanced areas in the arterial phase are risk factors for VETC positivity. Intratumoral necrosis may specifically indicate VETC structures where spherical tumor nests compress perivascular spaces, leading to localized tumor hypoperfusion and hypoxia. This study's findings align with previous research, showing significant differences in intratumoral necrosis between VETC-positive and VETC-negative groups.

Fan *et al*. [5] found a significant correlation between VETC positivity and non-ring-like, heterogeneous high enhancement in the arterial phase (P<0.05). This pattern relates to the hypervascularization and high heterogeneity of HCC, with VETC tumor clusters featuring endothelial cells forming vessels that supply nutrients and oxygen [16]. VETC has been confirmed to comprise functional perfused vessels [3]. Enhanced CT can illustrate the rich blood supply and intratumoral neovascularization. Previous studies also reported significant differences in intratumoral arteries among VETC-positive patients [5, 8, 13]. Yang *et al*. [17] demonstrated that intratumoral arteries and enhancement patterns are independent predictors of VM-HCC (VETC+/MVI-, VETC-/MVI+, VETC+/MVI+) subtypes, and are significantly associated with early and overall tumor recurrence. In this study, intratumoral arteries were observed in 63.6% (7/11) of VETC-positive cases, higher than the 42.9% (18/42) in VETC-negative cases, indicating a higher rate of angiogenesis in VETC-positive HCC. However, non-ring-like enhancement (72.7% *vs*. 81.0%) and marked heterogeneous enhancement (9.1% *vs*. 23.8%) were slightly lower in the VETC-positive group, potentially due to the small sample size and selection bias. Additionally, arterial phase CT values may serve as a predictive indicator for VETC positivity, a novel finding not previously reported in conventional imaging studies. Yang *et al*. [17] identified typical “fast-in, fast-out” enhancement patterns in VM-HCC. Yao *et al*. [18] developed a radiomics model based on preoperative CT to predict MVI, showing higher predictive values for arterial and delayed phases (AUC 0.79 and 0.80) compared to unenhanced and venous phases (AUC 0.75 and 0.73), indirectly supporting arterial phase CT values as potential predictors for VETC positivity.

Our study, however, has some limitations. Firstly, this was a single-center, retrospective study with a small sample size, warranting further expansion for robust conclusions. Secondly, selection bias was present, to comprehensively reflect the enhanced CT features of VETC-positive lesions; lesion size was not excluded. Larger sample sizes and further research are needed to validate the preoperative enhanced CT characteristics of VETC. Thirdly, other imaging modalities (*e.g*., MRI) may help determine the relative advantages and limitations of using enhanced CT for VETC prediction.

## CONCLUSION

In conclusion, contrast-enhanced CT, as one of the conventional and preferred examination methods for HCC, plays an important role in the diagnosis, staging and post-treatment evaluation. Combined with the CT value in the arterial phase of preoperative enhanced CT, intratumoral arteries and intratumoral necrosis can highly indicate VETC positivity. In the future, we will expand the sample size, pay attention to the changes of each indicator and the results of other imaging examinations, and further explore the importance of imaging indicators such as CT value of preoperative enhanced CT in the arterial phase, intratumoral artery and intratumoral necrosis.

## Figures and Tables

**Table 1:** Clinical characteristics of VETC-positive and VETC-negative HCC patients.

**Clinical Indicators**	**VETC-negative(42)**	**VETC-positive(11)**	**P-value**
Age	60.55±9.99	59.27±8.15	0.698
Gender (Male/Female)	36/6	10/1	0.651
ALB	39.89±4.17	40.35±5.12	0.761
TB	15.38±9.82	17.14±5.13	0.570
DB	5.92±7.12	5.15±1.87	0.727
AST	39.62±37.65	36±16.43	0.758
ALT	37.74±53.43	37.27±28.96	0.978
PT	11.75±1.05	11.92±0.66	0.613
TT	15.92±1.55	15.54±1.85	0.487
INR	1.08±0.17	1.06±0.06	0.788
AFP(20/20-400/400)	30/3/9	5/4/2	0.037
Recurrence (No/Yes)	27/15	3/8	0.027

**Table 2:** Pathological results comparison of VETC-positive and VETC-negative HCC patients (cases).

	**Histological Grade**			**MVI**		**Satellite Nodules**			**Neural Invasion**	
**Poor**	**Moderate**	**Well**	**Absent**	**Present**		**Absent**	**Present**	**Absent**	**Present**
**VETC-negative(42)**	3	33	6	25	17	37		5	11	0
**VETC-positive(11)**	2	9	0	4	7	8		3	41	1
**P-value**	0.258			0.17		0.205			0.605	

**Table 3:** Enhanced CT imaging characteristics comparison of VETC-positive and VETC-negative HCC patients (cases).

**Enhanced CT Imaging Features**	**VETC(-)**		**VETC(+)**		**P-value**
**Absent**	**Present**	**Absent**	**Present**
Tumor diameter (mm)	43.38±24.3		30.82±18.12		0.116
Intrahepatic growth	1(2.4)	41(97.6)	1(9.1)	10(90.9)	0.299
Irregular tumor margins	26(61.9)	16(38.1)	6(54.5)	5(45.5)	0.657
Peritumoral hepatic parenchymal enhancement	37(88.1)	5(11.9)	10(90.9)	1(9.1)	0.793
Mosaic structure	31(73.8)	11(26.2)	9(81.8)	2(18.2)	0.583
Non-ring-like arterial phase hyperenhancement	8(19.0)	34(81.0)	3(27.3)	8(72.7)	0.549
Marked heterogeneous enhancement	32(76.2)	10(23.8)	10(90.9)	1(9.1)	0.284
Non-peripheral washout	4(9.5)	38(90.5)	0(0.0)	11(100.0)	0.287
Absence of enhancing capsule	33(78.6)	9(21.4)	9(81.8)	2(18.2)	0.813
Enhancing/well-defined capsule	22(52.4)	20(47.6)	7(63.6)	4(36.4)	0.504
Intratumoral arteries	24(57.1)	18(42.9)	4(36.4)	7(63.6)	0.219
Intratumoral necrosis	21(50.0)	21(50.0)	10(90.9)	1(9.1)	0.014
Unenhanced CT values (HU)	44.76±6.06		44.27±4.73		0.805
Arterial phase CT values (HU)	75.07±18.37		67.82±16.55		0.240
Arterial phase enhancement ratio	0.68±0.39		0.54±0.37		0.282
Portal phase CT values (HU)	92.14±21.02		93.09±24.82		0.898
Portal phase enhancement ratio	1.05±0.41		1.1±0.48		0.705
Venous phase CT values (HU)	78.95±13.28		77.36±14.28		0.729
Venous phase enhancement ratio	0.77±0.28		0.75±0.23		0.769

**Table 4:** Multivariate analysis of factors influencing VETC positivity in HCC patients.

**Imaging Observational Indicators**	**B**	**SE**	**wald**	***P***	***OR(95%CI)***
Arterial Phase CT Values (HU)	-0.066	0.0301	4.752	0.029	0.937(0.883-0.993)
Intratumoral Arteries	2.246	0.974	5.313	0.021	9.452(1.4-63.823)
Intratumoral Necrosis	-4.378	1.468	8.897	0.003	0.013(0.001-0.223)

## Data Availability

The data and supportive information are available within the article.

## LIST OF ABBREVIATIONS

VETC: Vessels encapsulating tumor clusters
Alb: Albumin
AST: Aspartate aminotransferase
ALT: Alanine aminotransferase
TBil: Total bilirubin
DB: Direct bilirubin
PT: Prothrombin time
TT: Thrombin time
INR: International normalized ratio

## STANDARDS OF REPORTING

STROBE guidelines were followed.
